# Iron Deficiency Prolongs Seed Dormancy in *Arabidopsis* Plants

**DOI:** 10.3389/fpls.2017.02077

**Published:** 2017-12-08

**Authors:** Irene Murgia, Piero Morandini

**Affiliations:** Dipartimento di Bioscienze, Università degli Studi di Milano, Milan, Italy

**Keywords:** after-ripening, freshly harvested seeds, germination, iron deficiency, longevity, Reactive Oxygen Species, seed dormancy

## Abstract

The understanding of seed dormancy, germination and longevity are important goals in plant biology, with relevant applications for agriculture, food industry and also human nutrition. Reactive Oxygen Species (ROS) are key molecules involved in the release of dormancy, when their concentrations fall within the so called ‘oxidative window.’ The mechanisms of ROS distribution and sensing in seeds, from dormant to germinating ones, still need elucidation. Also, the impact of iron (Fe) deficiency on seed dormancy is still unexplored; this is surprising, given the known pro-oxidant role of Fe when in a free form. We provide evidence of a link between plant Fe nutrition and dormancy of progeny seeds by using different *Arabidopsis* ecotypes and mutants with different dormancy strengths grown in control soil or under severe Fe deficiency. The latter condition extends the dormancy in several genotypes. The focus on the mechanisms involved in the Fe deficiency-dependent alteration of dormancy and longevity promises to be a key issue in seed (redox) biology.

## Introduction

The still widespread micronutrient deficiencies in various developing countries, the increase in human population and the shifts in the diets taking place in many countries all urge for an increased production of staple foods of higher nutritional value (Murgia et al., 2012, 2013). A wider availability of elite genotypes of higher seed quality in terms of survival capacity and vigor, is an important component to achieve such goals. Best practices of seeds harvest and storage aimed at reducing loss in germination as well as deterioration of seeds are also needed. The understanding of the complex net of molecular players regulating seed vigor, viability and dormancy therefore represents a focus of primary importance in plant science (Grierson et al., 2011; Rajjou et al., 2012).

A seed is described as germinated when, upon imbibition, the integument is broken, the radicle starts to protrude and the embryonic axis elongates. The process of germination requires favorable conditions of moisture, light and temperature.

Dormant seed are, however, not able to germinate even under the most favorable conditions. Dormancy is indeed a protective mechanism which prevents germination in an unfavorable environment, such as germination out of season on the mother plant (pre-harvest sprouting), or at high temperature, which would kill germinated seedlings (Gubler et al., 2005; Kranner et al., 2010; Rajjou et al., 2012; Rodriguez et al., 2015; Nèe et al., 2017). Dormancy can be advantageous also in favorable environments since it spreads germination in time, thus reducing the competition among siblings and increasing the time available for seed dispersal. A complete loss of dormancy can be lethal, as evident from the viviparous mutants in many species (Chen et al., 2017); indeed, many wild species produce dormant seeds. On the other hand, seeds produced from various cultivated crop species show reduced dormancy; as an example, the trait of reduced dormancy has been selected during domestication of cereals and legumes to avoid erratic germination which would be detrimental for optimal productivity and grain use (Sugimoto et al., 2010; Rodriguez et al., 2015; Zinmeister et al., 2015; Sato et al., 2016; Ishibashi et al., 2017).

After a given period of dry storage, named ‘after-ripening,’ and/or through a period of moist chilling, named ‘cold stratification,’ the dormant seeds eventually loose their quiescent status and germinate. The mechanisms regulating this shift of status are complex, involving multiple semi-independent pathways involving hormones, dormancy-specific regulators and chromatin modifiers (Shu et al., 2016; Nèe et al., 2017). In particular, the balance between the two hormones abscisic acid (ABA) and gibberellins (GA) is a central node of regulation of dormancy; ABA and GA act, respectively, as inhibitor and promoters of germination (Piskurewicz et al., 2008; Lee et al., 2010; Rajjou et al., 2012; Shu et al., 2016). ABA is synthesized, in *Arabidopsis* seeds, in the endosperm, i.e., in the cell layer forming the inner part of the seed coat, which also comprises an external layer of dead cells named testa; germination starts with testa rupture and is completed with endosperm rupture; notably, the removal of the endosperm, but not of the seed testa, is sufficient to induce germination (Lee et al., 2010). Dormant seeds have the capacity to maintain, upon imbibition, high levels of ABA.

Reactive Oxygen Species (ROS) are key players in seed dormancy and longevity (Rajjou et al., 2012) and are produced during imbibition as well as during the dry state (Ventura et al., 2012), by enzymatic and non-enzymatic reactions. Inner structures are hypoxic environments, and seeds limit oxygen diffusion to extend their viability; indeed, ROS can act as cytotoxic agents by damaging nuclei acids, proteins and lipids; a role of hydrogen peroxide (H_2_O_2_) in seed aging has been thoroughly described by various research groups (Kumar et al., 2015; Wojtyla et al., 2016 and references therein). However, below a given ROS concentration threshold, seed would not germinate; a ‘ROS concentration window’ necessary for germination has been proposed: below a certain concentration the seeds remain dormant whereas, above a higher concentration, they start to loose their viability because of damage (Bailly et al., 2008; Leymarie et al., 2012; Ventura et al., 2012). H_2_O_2_ is implicated in the release of dormancy through endosperm loosening, its probable oxidation of reserve proteins and their mobilization, and its interaction with the hormones ABA and GA as well as with other signaling molecules, such as nitric oxide (NO) (Kumar et al., 2015; Wojtyla et al., 2016). Also, a selective oxidation of targeted mRNAs and proteins acts as positive mechanism for germination (Bazin et al., 2011; El-Maarouf-Bouteau et al., 2013). Such findings confirm what already established since years in both animal and plant cells, i.e., that ROS are not mere cytotoxic agents causing oxidative stress and damage of macromolecules but, at certain concentrations, they are in fact important signaling molecules involved in the so-called “redox biology” (Schieber and Chandel, 2014; Kumar et al., 2015; Mittler, 2017).

The shift of status from quiescent to active does not always represent, for an embryo, the ability to complete germination, since the seed coat might exert a mechanical resistance to rupture; this phenomenon is described as ‘coat-imposed’ dormancy and it involves the regulation of genes expression in the endosperm region surrounding the radicle tip (Nonogaki, 2014). According to a recent hypothesis, the induction of such genes is caused by mechanosensing (Dekkers et al., 2013; Nonogaki, 2014).

Experimental evidences supported, in the past, the idea that reduced seed longevity correlated with reduced dormancy; this notion has been recently challenged by a study of natural variation for seed longevity, in which the two traits are in fact negatively correlated, i.e., high levels of dormancy correlate with low storability (Nguyen et al., 2012).

## Link Between Fe Nutritional Status, Seed Dormancy and Seed Longevity

The impact of the plant iron (Fe) nutritional status in terms of yield and nutritional value of edible parts of staple crops inspired, since decades, the research on Fe uptake from soil, its distribution into the various plant organs, and its trafficking, storage and metabolism. A variety of agronomical, physiological, biochemical, genetic and “-omic” approaches have been applied and provided a detailed view on Fe nutrition mechanisms in plants (Briat et al., 2009; Morrissey and Guerinot, 2009; Roschzttardtz et al., 2009; Ramirez et al., 2011; Kobayashi and Nishizawa, 2012, 2014; Murgia et al., 2012, 2013; Vigani et al., 2013; Grillet et al., 2014; Brumbarova et al., 2015; Tsai and Schmidt, 2017).

Iron, when in a free, redox-active form, can catalyze the Fenton reaction, which produces hydroxyl radicals (OH⋅) from the less reactive H_2_O_2_ and ion superoxide (O_2_**^-⋅^**). The Fe metabolic status of a plant is therefore tightly connected with its ‘ROS status’ (Ravet and Pilon, 2013).

The diffusion kinetics of the different ROS species in seeds depend, among others, on seed status, whether desiccated or hydrated; this, in turn, influences how seeds can sense the different ROS; a detailed discussion of such a point can be found in Kumar et al. (2015).

Taken together, these premises suggest that changes in the Fe nutritional status of a mother plant might affect the dormancy of progeny seeds, as well as their longevity; indeed, the altered localization, concentration and, most important, redox state of Fe in seeds can exert profound effects in terms of change of balance among the different ROS species and, hence, on ROS diffusion and sensing.

The link between nitrate and seed dormancy is already established: increasing concentrations of nitrate in the growth medium of *Arabidopsis* plants cause a reduction of dormancy, in the progeny seeds (Alboresi et al., 2005). Further results obtained by these authors support the hypothesis that the nitrate accumulated in seeds is responsible for the perturbation of dormancy and that nitrate acts as a signal molecule (Alboresi et al., 2005). The exposure of *Arabidopsis* plants to low nitrate supply after flowering, decreases the germination rate of produced seeds, although no significant effects on dormancy are observed, after exposure to high nitrate supply (He et al., 2014). Phosphate can also slightly affect seed dormancy (He et al., 2014). The effect of Fe deficiency on seed dormancy has never been investigated, so far.

## Fe Deficiency Affects Seed Dormancy

*Arabidopsis* ecotypes and mutants of various dormancy strengths are useful for exploring the link between Fe and dormancy; among the wt ecotypes, the widely used Col (Columbia) has intermediate dormancy, whereas Cvi (Cape Verde Island) is highly dormant and Bur (Burren, Ireland) is non-dormant (Footitt et al., 2013). Among the *Arabidopsis* mutants, ABA2 mutant overexpressing the ABA2 biosynthetic gene shows around 45% increase in ABA content whereas the *aba2* mutant has approximately 27% residual ABA levels, compared to their wt Col; their dormancy is, respectively, longer and shorter than their wt Col (Lin et al., 2007).

**FIGURE 1:**
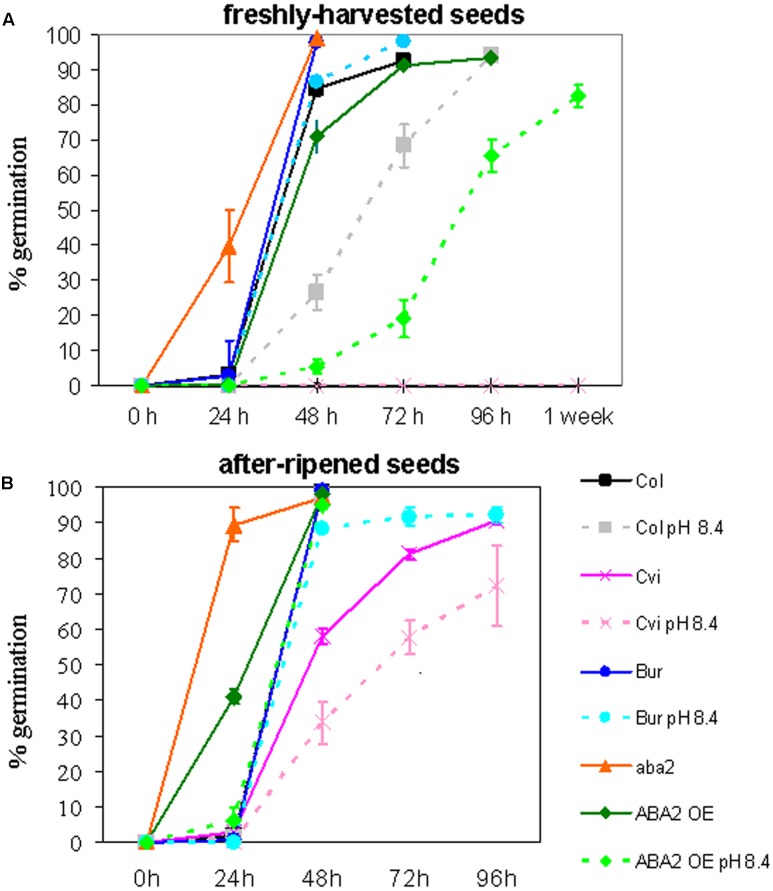
*Arabidopsis* Col (Col-0), Cvi, Bur, *aba2*, and ABA2OE seeds collected from plants grown in control or in alkaline soil (pH 8.4) were tested for germination on moist paper at 22°C, 70 μmol photons m^-2^ sec^-1^, 16 h/8 h light/dark. **(A)** Germination of freshly harvested seeds after 24, 48, 72, 96 h or 1 week from imbibition, **(B)** germination of seeds after 5 months of storage at room temperature (after ripened seeds) after 24, 48, 72 or 96 h from imbibition. Each point is the mean value ± SE of % germinated seeds of at least six plates, with at least one hundred seeds each. **(B)** The line corresponding to Bur (in blue) completely overlaps the lines corresponding to Col control and Col pH 8.4.

Col (Col-0), Cvi, Bur, *aba2*, and ABA2OE were grown in control soil or under severe Fe deficiency, in alkaline soil (pH 8.4; soil prepared as described in Murgia et al., 2015). All the plants produced seeds, with the exception of *aba2* grown in alkaline soil, which could not reach maturity. Dormancy of the ’freshly harvested’ seeds and of the ‘after-ripened’ ones (5 months old) was measured (**Figures 1A,B**, respectively). Germination of freshly harvested seeds of intermediate dormancy, such as ABA2OE and Col, shows that growth of the mother plant under Fe deficiency prolongs the dormancy of the seeds produced; ABA2OE and Col seeds show indeed a germination delay when their mother plants are grown in alkaline soil with respect to control soil (**Figure 1A**). Such an effect could not be observed in Cvi or in Bur ecotypes, since freshly harvested Cvi seeds remain dormant whereas Bur always germinate, regardless of the soil condition of their mother plant (**Figure 1A**). However, the ’after-ripening’ allows the Cvi seeds to partially weaken their dormancy and hence to unmask the effect of alkaline soil on seed germination (**Figure 1B**).

These results obtained with different *Arabidopsis* lines of various dormancy strength, when grown under Fe deficiency, are compatible with the working hypothesis, i.e., that Fe deficiency alters establishment of dormancy in progeny seeds.

A negative correlation between seed dormancy and viability has been recently described in *Arabidopsis* (Nguyen et al., 2012), in *Eruca sativa* (Barazani et al., 2012; Hanin et al., 2013) and in *Galinsoga parviflora* and *G. quadriradiate* (De Cauwer et al., 2013). In a study conducted to investigate transgenerational Fe deficiency stress memory in *Arabidopsis*, it turned out that growth of more than one generation under mild Fe deficiency (soil at pH 7.7) had a positive impact on the longevity of the seeds produced (Murgia et al., 2015). Therefore, the negative correlation between dormancy and longevity, mentioned above, might not be conserved under Fe deficiency.

These results encourage to proceeding further with the analysis of the impact of Fe deficiency on seed dormancy and viability. The testing of a large-scale collection of ’freshly harvested’ seeds from various lines of intermediate dormancy, when grown under Fe-deficiency is needed. Production of such collection is quite demanding, since plants produce few seeds when grown under severe Fe deficiency and the collection of freshly harvested seeds implies accurate inspection of each silique on a daily basis, to guarantee a genuine fresh harvest.

Nevertheless, the suggested analysis could allow to confirm, at large scale, the effect of Fe nutrition on seed dormancy. Such a finding might represent the premise to proceed further in the detailed search of the molecular mechanisms underlying such a phenomenon; it would also enable to measure, at large scale, the natural longevity of the progeny seeds produced from mother plants exposed to different Fe nutritional conditions and its correlation with dormancy.

## Concluding Remarks

The alteration in dormancy observed under Fe deficiency might be the outcome of the interplay between Fe and ROS occurring during the time-window spanning from seed maturity to germination. Tackling the issue of the extent and of the mechanisms involved in the Fe-deficiency-dependent alteration of dormancy and longevity can represent an important step forward in seed (redox) biology.

## Author Contributions

IM planned and performed the experiments. IM and PM analyzed and discussed the results obtained. IM wrote the manuscript, with contributions by PM.

## Conflict of Interest Statement

The authors declare that the research was conducted in the absence of any commercial or financial relationships that could be construed as a potential conflict of interest.
